# Cementless vs. Cemented Total Knee Arthroplasty: Reduced Operative Time with Comparable Perioperative Safety—A Retrospective Cohort from a Tertiary Care Center

**DOI:** 10.3390/jcm14217890

**Published:** 2025-11-06

**Authors:** Marco Basso, Giuseppe Anzillotti, Luca Ruosi, Elizaveta Kon, Marco Minelli, Enrico Arnaldi

**Affiliations:** 1 IRCCS Humanitas Research Hospital, Via Manzoni 56, 20089 Rozzano, Milan, Italy; marco.basso@humanitas.it (M.B.); luca.ruosi@humanitas.it (L.R.); elizaveta.kon@humanitas.it (E.K.); marcomariaminelli@gmail.com (M.M.); enrico.arnaldi@humanitas.it (E.A.); 2 Department of Biomedical Sciences, Humanitas University, Via Rita Levi Montalcini 4, 20072 Pieve Emanuele, Milan, Italy

**Keywords:** arthroplasty, replacement, knee, operative time, hemoglobins, total knee arthroplasty

## Abstract

**Background:** Total knee arthroplasty (TKA) is a widely performed and highly successful procedure, with cemented fixation historically considered the standard. Advances in implant design have renewed interest in cementless fixation, which shows comparable survivorship and increasing use, particularly in younger patients. Evidence on perioperative outcomes remains mixed, prompting this study to compare hemoglobin decline and operative time between cemented and cementless TKA of the same design. **Methods:** This monocentric retrospective cohort study included consecutive patients undergoing primary TKA between 2019 and 2021, divided into cemented and cementless groups. Inclusion criteria were primary osteoarthritis, age > 45 years, hemoglobin > 13 g/dL, ferritin > 100 ng/mL, and complete perioperative hemoglobin data (preoperative, PostOperativeDay (POD)1, 3, 5). Primary outcomes were operative time and hemoglobin trajectory, analyzed using multivariable regression and mixed-effects models adjusted for age, sex, and implant design. Propensity score matching was performed as a sensitivity analysis. **Results:** A total of 123 TKAs were analyzed (63 cementless, 60 cemented). Cementless TKA had a shorter operative time than cemented (72.0 ± 12.0 vs. 79.8 ± 15.1 min; 95% CI −12.2 to −2.8; *p* < 0.01). Cementless fixation was associated with significantly shorter operative time (72.0 ± 11.8 vs. 79.8 ± 15.1 min, *p* < 0.01), a difference that remained significant after multivariable adjustment and propensity score matching. Postoperative hemoglobin declined in both groups, with no significant unadjusted between-group differences at any timepoint. In adjusted mixed-effects models, cementless TKA was associated with a slightly greater hemoglobin decline compared with cemented TKA, with mean adjusted differences of −0.56 g/dL on POD1 (95% CI [−0.95, −0.17]), −0.53 g/dL on POD3 (95% CI [−0.91, −0.14]), and −0.34 g/dL on POD5 (95% CI [−0.64, −0.04]). However, this was not clinically relevant, as no transfusions or anemia-related complications occurred. **Conclusions:** Cementless TKA was associated with reduced operative time compared with cemented fixation, an effect robust to adjustment and propensity score matching. These conclusions apply to a selected, relatively non-anemic cohort. Although hemoglobin decline was slightly greater with cementless fixation, the difference was small and not clinically meaningful.

## 1. Introduction

Total knee arthroplasty (TKA) involves replacing the entire femorotibial joint and is one of the most commonly performed and successful surgical procedures, effectively restoring joint function [1].

Projections show that the demand for such procedure will keep increasing worldwide over the next decades [2], mostly due to the ageing population and the increasing rate of obesity [3]. Cemented fixation has historically become the standard in primary TKA, largely due to its reliable early stability and wide applicability. However, improvements in implant geometry, porous coatings, and surface technologies have renewed interest in cementless fixation for primary TKA. Recent reviews and registry-based analyses indicate that modern cementless constructs achieve survivorship comparable to—and in some cases exceeding—that of cemented designs, while providing similar patient-reported outcomes [4,5]. Alongside these technical advances, national registries and observational cohorts report a steady rise in cementless TKA utilization over the past decade, especially among younger and higher-demand patients, where durable biologic fixation is advantageous. In the U.S., for instance, registry analyses show cementless use increasing from approximately 4% in 2015 to nearly 15% in 2021, reflecting similar international trends [6]. Despite this momentum, evidence regarding short-term perioperative outcomes remains mixed. Multiple matched and propensity-adjusted studies suggest that, when modern blood-management protocols are employed, cementless fixation is not associated with clinically meaningful increases in blood loss, hemoglobin decline, or transfusion rates [7,8]. Conversely, other studies and reviews note small and inconsistent differences in perioperative hemodynamic measures between cemented and cementless fixation, differences unlikely to affect early clinical outcomes [9]. In terms of efficiency, analyses of same-design implants have shown shorter operative time with cementless TKA—attributed to eliminating cement preparation, application, and pressurization—without compromising early survivorship [10]. Furthermore, mid-term randomized and cohort studies suggest that modern cementless platforms provide early clinical outcomes comparable to cemented fixation [11,12]. Hence, the present retrospective study aimed to compare early postoperative outcomes, specifically hemoglobin (Hb) levels and operative time, between cemented and cementless TKA of the same design. We hypothesized that cementless fixation would reduce operative time but result in greater blood loss.

## 2. Materials and Methods

This is a monocentric retrospective cohort study on a consecutive series of patients who underwent primary total knee arthroplasty (TKA) between 2019 and 2021 The study was approved by the independent Institutional Ethics Committee of the IRCCS Humanitas Research Hospital (protocol number 618/17; date 18 December 2017). Written informed consent was obtained for all the patients included in the present study. Patients were divided into two groups according to components fixation technique: cemented and cementless TKA. Inclusion criteria were primary knee osteoarthritis, age > 45 years, hemoglobin > 13 g/dL, ferritin > 100 ng/mL, hence yielding a relatively non-anemic cohort and availability of complete perioperative hemoglobin (Hb) data at baseline (preoperative), postoperative day (POD) 1, POD 3, and POD 5. Exclusion criteria included revision procedures, inflammatory arthropathies, concomitant patellar resurfacing, coagulopathies, rheumatological diseases, hemoglobin levels ≤ 13 g/dL, ferritin ≤ 100 ng/mL, use of anticoagulant or antiplatelet therapy, or missing perioperative records.

### 2.1. Surgical Technique

All procedures were performed by a single fellowship-trained high-volume arthroplasty surgeon at a tertiary care arthroplasty center. All patients underwent a standardized medial parapatellar approach, and the Vanguard Knee System (Zimmer Biomet, Warsaw, IN, USA) was used in every case. Both cruciate-retaining (CR) and posterior-stabilized (PS) designs were used, according to intraoperative assessment of joint stability. The decision to perform cemented or cementless fixation was made intraoperatively based on bone quality, implant stability, and reduction in deformity. Perioperative antibiotic prohylaxis was carried out with one shot of 2 g cefazolin or 600 mg clindamycin for patients who reported to be allergic to beta-lactams as per institutional protocol. Spinal anesthesia was administered in the L3–L4 subarachnoid space using a 25G Whitacre needle with the patient in the lateral decubitus position (operative limb uppermost). A dose of 2.5 mL of 0.5% levobupivacaine was injected. During the surgery patients were given slight sedation with 1 or 2 mg of midazolam. A pneumatic tourniquet was applied in all the cases and inflated up to 250 mmHg, deflated during hemostasis, and re-inflated during cement fixation in the cemented group. Systolic blood pressure was consistently maintained below 120 mmHg. Tranexamic acid was administered both intravenously (1 g) before the procedure and intra-articularly (3 g) injected immediately after the surgical closure of the capsule, according to institutional protocol. Closed-suction drains were not used in any patient. Antithromboembolic prophylaxis consisted of enoxaparin 4000 IU subcutaneously, initiated 12 h after spinal anesthesia and continued for 1 month. Patients were assisted to stand and begin ambulation on POD 0. Only paracetamol and opioids were administered postoperatively; no NSAIDs were used. Blood transfusion was indicated for patients with hemoglobin levels < 8 g/dL or in cases of symptomatic anemia, according to institutional protocol.

### 2.2. Data Collection and Outcomes

Demographic variables (age, sex), implant design (CR vs. PS), operative time (minutes), and perioperative Hb values were extracted from medical records. Operative time was measured from skin incision to wound closure. The primary outcomes were operative time and perioperative Hb trend. Secondary outcome was ΔHb (difference between preoperative and postoperative Hb at each timepoint).

### 2.3. Statistical Analysis

Continuous variables were summarized as mean ± standard deviation (SD) or median with interquartile range (IQR), and categorical variables as frequencies and percentages. Baseline group balance was assessed using standardized mean differences (SMD) and chi-square tests for categorical variables. Between-group comparisons were performed with Welch’s *t*-test for continuous variables and chi-square tests for categorical variables.

Operative time was analyzed using a multivariable linear regression model with fixation method (cemented vs. cementless) as the main predictor, adjusting for age, sex, and implant design.

Hemoglobin trajectories were analyzed after reshaping the dataset into long format, using a linear mixed-effects model with random intercepts for each patient and fixed effects for group, time (preoperative, POD1, POD3, POD5), and their interaction. Models were adjusted for age, sex, and implant design. The primary effect of interest was the group × time interaction, testing whether hemoglobin decline differed between fixation methods. Estimated marginal means with 95% confidence intervals (CIs) were calculated for each group and timepoint. No formal a priori power calculation was performed due to the retrospective design.

To mitigate potential confounding by indication, a propensity score analysis was conducted. Propensity scores were estimated via logistic regression including age, sex, and implant design, followed by 1:1 nearest-neighbor matching without replacement. Analyses for operative time and hemoglobin trajectories were repeated in the matched cohort as a sensitivity analysis. Post-match covariate balance was evaluated using standardized mean differences (SMDs) and displayed in a Love plot; SMDs < 0.10 were considered well balanced.

All analyses were performed using IBM SPSS Statistics Version 25 (IBM Corp., Armonk, NY, USA). A *p*-value < 0.05 was considered statistically significant.

## 3. Results

### 3.1. Patient Characteristics

A total of 123 primary TKAs were analyzed: 63 cementless and 60 cemented. Mean age did not differ significantly between groups (67.9 ± 8.0 vs. 68.9 ± 7.5 years, *p* = 0.50). Implant design distribution varied, with cruciate-retaining (CR) prostheses more common in the cementless group (54.0% vs. 31.7%, *p* = 0.02). Sex distribution also differed, with a higher proportion of males in the cementless group (66.7% vs. 38.3%, *p* < 0.01). Preoperative hemoglobin was slightly higher among cementless patients (14.4 ± 1.2 vs. 13.8 ± 1.3 g/dL, *p* = 0.02). Perioperative protocols adopted for both groups are listed in Supplementary Table S1.

### 3.2. Operative Time

**Figure 1:**
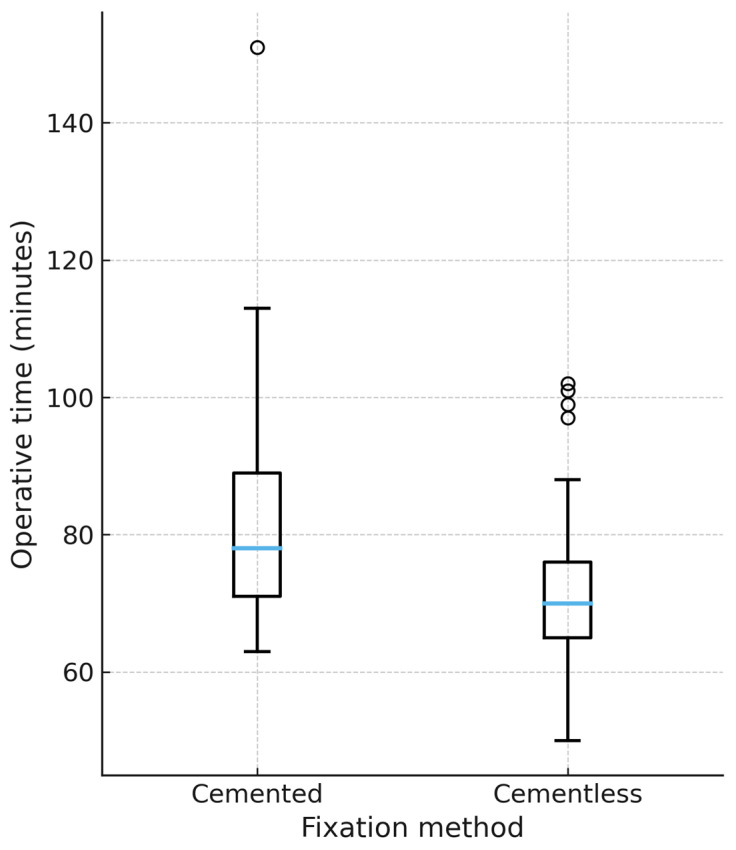
Operative time by fixation method (propensity-matched cohort). Boxplot representation of operative time (minutes) in cemented and cementless TKA after 1:1 propensity score matching on age, sex, and implant design. Median, interquartile range, and outliers are displayed. Cementless TKA was associated with a significantly shorter operative time (*p* < 0.01). Only matched patients are shown (60 = cemented, 60 = uncemented). Unmatched cases were excluded from the plot.

Mean operative time was significantly shorter in the cementless group (72.0 ± 11.8 min) compared with the cemented group (79.8 ± 15.1 min), with a mean difference of 7.8 min (*p* < 0.01, Welch’s *t*-test). In a multivariable regression model adjusting for age, sex, and implant design, cementless fixation remained independently associated with reduced operative time (β −7.5 min, 95% CI −12.2 to −2.8, *p* < 0.01). This association persisted after propensity score matching (1:1 on age, sex, and design), as illustrated in Figure 1.

### 3.3. Hemoglobin Trajectory

**Figure 2:**
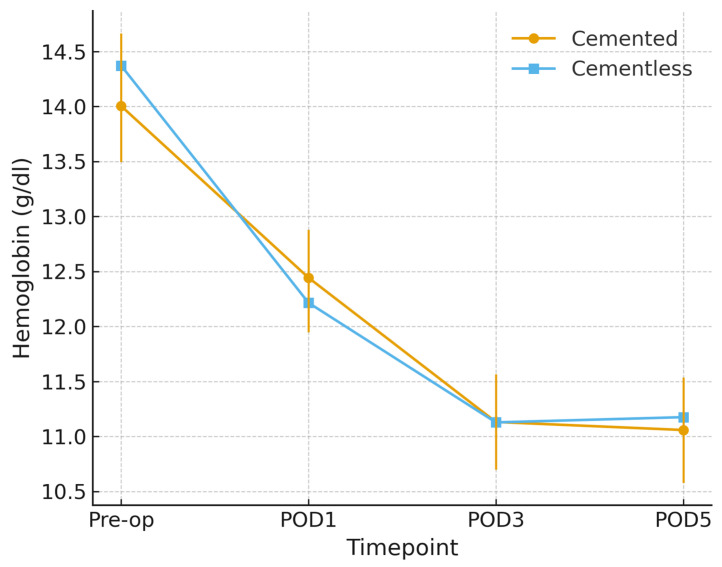
Hemoglobin trajectory by fixation (propensity-matched cohort). Mean hemoglobin values, in g/dL (±95% CI) from preoperative baseline to postoperative day (POD) 5 in cemented and cementless TKA after 1:1 propensity score matching on age, sex, and implant design. Both groups show a postoperative decline; cementless TKA exhibits a slightly steeper reduction across timepoints.

Postoperative hemoglobin declined in both groups. In unadjusted comparisons, no significant between-group differences were observed at any timepoint (all *p* > 0.80). However, in a mixed-effects model adjusted for age, sex, and implant design, the group × time interaction reached statistical significance (*p* < 0.05). Cementless TKA was associated with a slightly greater hemoglobin decline compared with cemented TKA, with mean adjusted differences of −0.56 g/dL on POD1 (95% CI [−0.95, −0.17]), −0.53 g/dL on POD3 (95% CI [−0.91, −0.14]), and −0.34 g/dL on POD5 (95% CI [−0.64, −0.04]). These differences were small in magnitude and did not result in clinically relevant events, as no transfusions, according to our institutional protocol, nor anemia-related complications occurred. Estimated marginal means are shown in Figure 2.

### 3.4. Propensity Score Analysis

**Figure 3:**
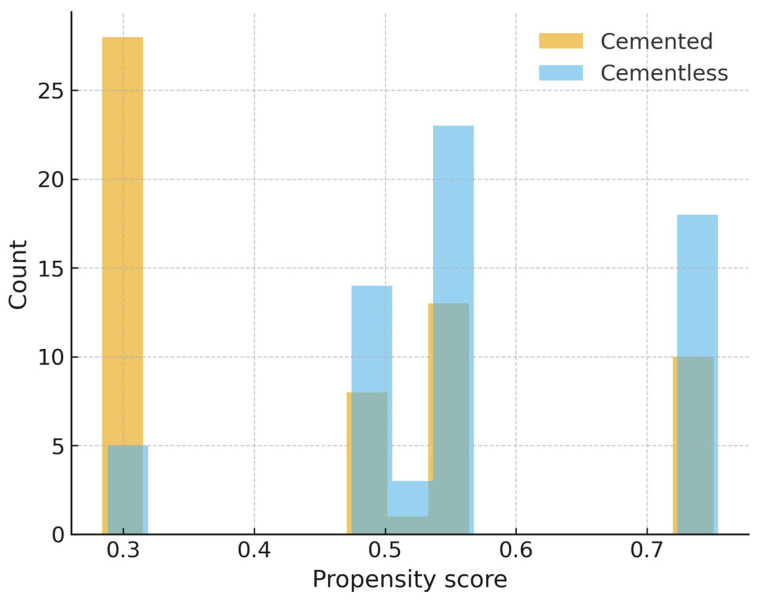
Propensity score distribution before matching. Histogram of estimated propensity scores for cemented and cementless TKA prior to matching, showing baseline imbalance between groups.

**Figure 4:**
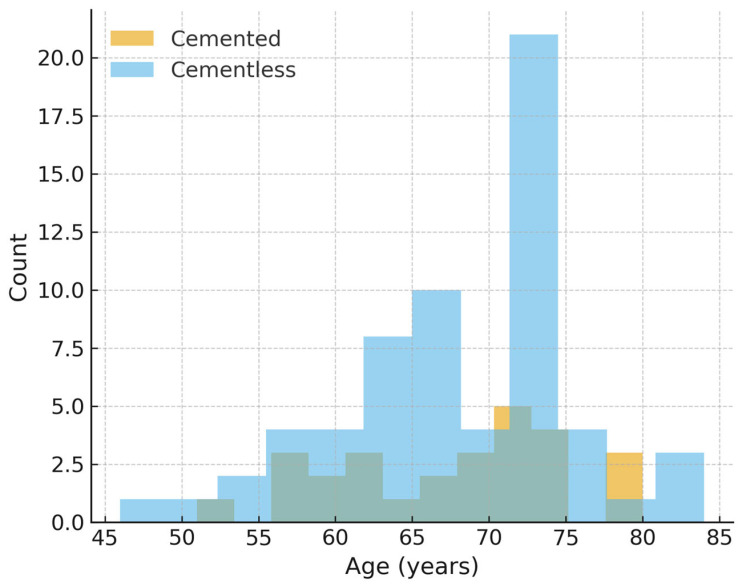
Age distribution after propensity score matching. Age distribution after 1:1 propensity score matching. Full post-match balance (age, sex, type of implant) is summarized in Table 1 and in the Love plot (Supplementary Figure S1).

Propensity score distributions demonstrated moderate baseline imbalance prior to matching (Figure 3). Matching improved covariate balance, particularly for age and implant design (Figure 4). Findings for operative time and hemoglobin decline in the matched cohort were consistent with the primary analyses.

Post-match covariate balance and key outcomes are summarized in Table 1 (age, sex, CR/PS, operative time, ΔHb at POD1/3/5; SMDs pre- and post-match) and visualized in a Love plot (Supplementary Figure S1).

## 4. Discussion

The main findings of this study are that cementless TKA was associated with a significantly shorter operative time compared with cemented TKA, and with a slightly greater postoperative hemoglobin decline. However, the difference in hemoglobin trajectory was small (<0.6 g/dL) and not clinically meaningful. Cementless fixation reduced operative time by approximately 7–8 min, an effect that remained significant after adjustment for confounders and in the propensity score–matched cohort.

This finding of reduced operative time with cementless fixation is consistent with prior reports attributing this advantage to the elimination of cement mixing, application, and pressurization steps [10]. Similarly, Lawrie et al. observed an average of 11.6 min longer operative time for cemented compared to cementless TKA with implants of similar design, translating into an estimated additional cost of $418 per procedure [13]. Registry and review data further highlight that cementless fixation can improve surgical efficiency without compromising survivorship [14]. Even in case of robotic-assisted TKA, recent reports suggest that cementless constructs may preserve this advantage even in computer-assisted workflows, where cementing remains an additional time-consuming step [15]. Indeed, although a difference of less than 10 min may appear modest, in high-volume centers this efficiency can translate into meaningful improvements in operating room throughput and cost-effectiveness [16]. Future studies incorporating a health-economic perspective would be valuable to quantify the potential cost–benefit of cementless fixation. Although shorter operative times have been associated with lower complication risk in prior literature [17], our study did not measure periprosthetic joint infection and cannot make causal inferences regarding infection risk.

Both cemented and cementless groups experienced expected postoperative decreases in hemoglobin. Mixed-effects modeling identified a slightly greater decline in the cementless group, although the absolute difference was small and of limited clinical relevance. Importantly, the absence of transfusions occurred within a patient blood-management framework that triggers transfusion only at Hb < 8 g/dL or in symptomatic anemia, reinforcing the clinical irrelevance of the small adjusted differences in early Hb decline. This difference may in part reflect variations in tourniquet management, as cemented procedures typically require re-inflation during cement pressurization, which is a step avoided with cementless fixation. Previous studies on the topic investigated the hemodynamic outcomes of both cemented and cementless TKA and found no significant differences in total blood loss and transfusion rate [7,8,18]. The slightly greater hemoglobin decline observed with cementless TKA may reflect persistent bleeding from exposed cancellous bone surfaces, whereas in cemented fixation the pressurized cement likely provides a tamponade effect that limits intraosseous bleeding [19,20]. Consistent with this mechanism, in our standardized pathway only cemented procedures involved tourniquet re-inflation during cement pressurization (Supplementary Table S1), a step that can transiently augment tamponade of cancellous bone and plausibly contribute to the modestly smaller adjusted Hb drop observed with cemented fixation. Alternatively, differences in perioperative hemostatic control—such as local cauterization, timing of tranexamic acid administration, or tourniquet re-inflation during cement pressurization—could contribute to small but measurable differences in early postoperative hemoglobin trends [9,21]. Nevertheless, as several previous well-designed studies show no clinically relevant difference in blood loss and transfusion rates, our results suggest these mechanistic concerns do not typically translate into worse patient outcomes [7,8]. Indeed, while tourniquet use has been shown to reduce intraoperative blood loss, transfusion rate, and operative time, it does not significantly influence postoperative or total blood loss, transfusion volume, or length of stay [22,23]. Tourniquet use has been linked to slower early functional recovery, with RCTs and reviews reporting worse early quadriceps function, higher thigh/knee pain, and delays in early rehab milestones when a tourniquet is used [24,25]. Moreover, it also carries a higher risk of thromboembolic and minor wound complications, including increased deep vein thrombosis (DVT) and skin/tension blisters, even when total blood loss is unchanged [26,27].

Strengths of this study include the use of a single implant design and standardized surgical and perioperative protocols, which minimize variability between groups. All procedures were performed by a single fellowship-trained high-volume arthroplasty surgeon in a tertiary center, enhancing internal validity. Furthermore, perioperative hemoglobin was systematically assessed across multiple timepoints, and robust statistical methods, including propensity score matching, were applied to mitigate confounding. These features strengthen the reliability of our findings regarding operative efficiency and short-term safety of cementless versus cemented fixation.

However, this study has several limitations. First, the retrospective design carries a risk of residual confounding, despite the use of propensity score matching. In fact, it remains subject to selection bias since the decision to cement was made intraoperatively (e.g., bone quality, stability, reducibility) and such factors were not fully captured, leaving potential residual confounding. Additional clinical covariates such as BMI, ASA status, or preoperative alignment were not available and could not be included in the propensity model, which may leave residual confounding. Hospital stay was not recorded, and no transfusions occurred (95% CI 0.0–2.9%). Consequently, our conclusions regarding perioperative safety are limited to early hematologic outcomes. Future prospective studies with complete perioperative data are warranted. While the sample size was sufficient to detect differences in operative time, it may be underpowered to assess rare perioperative complications such as transfusion requirements or thromboembolic events. Larger, multicenter prospective studies are warranted to validate these findings and to provide a more comprehensive assessment of the comparative safety of cemented and cementless fixation. Another important limitation is the lack of direct measurement of intraoperative blood loss (e.g., suction volume, swab weight), which prevents precise quantification of bleeding differences between fixation types. Although perioperative hemoglobin trends offer an indirect estimate, future prospective studies should incorporate standardized intraoperative blood loss measurement to more accurately compare the hemostatic profile of cemented and cementless TKA. The absence of clinical and functional outcome measures (PROMs) such as the Knee Society Score (KSS), Oxford Knee Score (OKS), or range of motion (ROM) represents another limitation of this study. While our analysis was focused on perioperative parameters to ensure standardized comparison, future follow-up investigations are planned to assess medium- and long-term clinical and functional outcomes in this cohort. The monocentric and single-surgeon setting may also limit the generalizability of our findings. Future prospective, multicenter randomized trials with longer follow-up are warranted to confirm these results and to explore potential long-term differences in implant survival, functional outcomes, and cost-effectiveness between cemented and cementless fixation.

## 5. Conclusions

Cementless TKA was associated with shorter operative time compared with cemented fixation, while peri-operative Hb differences were small and clinically negligible within a standardized pathway. These conclusions apply to a selected, relatively non-anemic cohort. Operational benefits in high-volume centers are plausible; prospective multicenter studies—including PROMs, direct blood-loss metrics, and long-term survivorship—are warranted.

## Figures and Tables

**Table 1:** Baseline characteristics and key outcomes before and after 1:1 propensity score matching (PSM) between cemented and cementless TKA. Propensity variables were age, sex, and implant design (CR/PS); matching used nearest neighbor without replacement. Standardized mean differences (SMDs) are reported only for propensity variables to document balance; values < 0.10 indicate good balance (≤0.20 acceptable). Operative time and ΔHb at POD1/3/5 are shown for clinical context and were not used in the matching algorithm. Values are mean ± SD unless otherwise specified.

Section	Variable	Cemented	Cementless	SMD
Pre-match	Age (years)	69.3 ± 8.1	68.0 ± 7.8	−154
Pre-match	Male, n (%)	21 (36.2%)	41 (66.1%)	599
Pre-match	CR design, n (%)	19 (32.8%)	33 (53.2%)	413
Pre-match	Operative time (minutes)	79.5 ± 15.3	72.2 ± 11.9	
Pre-match	ΔHb POD1 (g/dL)	1.6 ± 0.8	2.1 ± 0.7	
Pre-match	ΔHb POD3 (g/dL)	2.7 ± 1.0	3.2 ± 1.0	
Pre-match	ΔHb POD5 (g/dL)	2.9 ± 1.1	3.2 ± 1.0	
Post-match	Age (years)	69.3 ± 8.1	67.9 ± 8.0	−165
Post-match	Male, n (%)	21 (36.2%)	38 (65.5%)	586
Post-match	CR design, n (%)	19 (32.8%)	31 (53.4%)	418
Post-match	Operative time (minutes)	79.5 ± 15.3	72.3 ± 12.2	
Post-match	ΔHb POD1 (g/dL)	1.6 ± 0.8	2.2 ± 0.7	
Post-match	ΔHb POD3 (g/dL)	2.7 ± 1.0	3.3 ± 1.0	
Post-match	ΔHb POD5 (g/dL)	2.9 ± 1.1	3.2 ± 1.0	

TKA, total knee arthroplasty; CR, cruciate-retaining; PS, posterior-stabilized; Hb, hemoglobin; POD, postoperative day; PSM, propensity score matching; SMD, standardized mean difference.

## Data Availability

The original contributions presented in this study are included in the article/Supplementary Materials. Further inquiries can be directed to the corresponding author.

## Supplementary Materials

The following supporting information can be downloaded at: https://www.mdpi.com/article/10.3390/jcm14217890/s1, Figure S1. Love plot of covariate balance before and after 1:1 propensity score matching (age, sex, implant design [CR]). Dashed vertical lines at 0.10 and 0.20 denote common balance thresholds; Table S1. Standardized peri-operative protocol common to cemented and cementless TKA.
