# Supplementary material for: Cementless vs. Cemented Total Knee Arthroplasty: Reduced Operative Time with Comparable Perioperative Safety—A Retrospective Cohort from a Tertiary Care Center
Source: J Clin Med. 2025 Nov 6;14(21):7890. doi: 10.3390/jcm14217890 (PMC12608410; doi:10.3390/jcm14217890)
Supplement: Supplementary file 1 [file jcm-14-07890-s001.zip › jcm-3939065-supplementary.pdf]

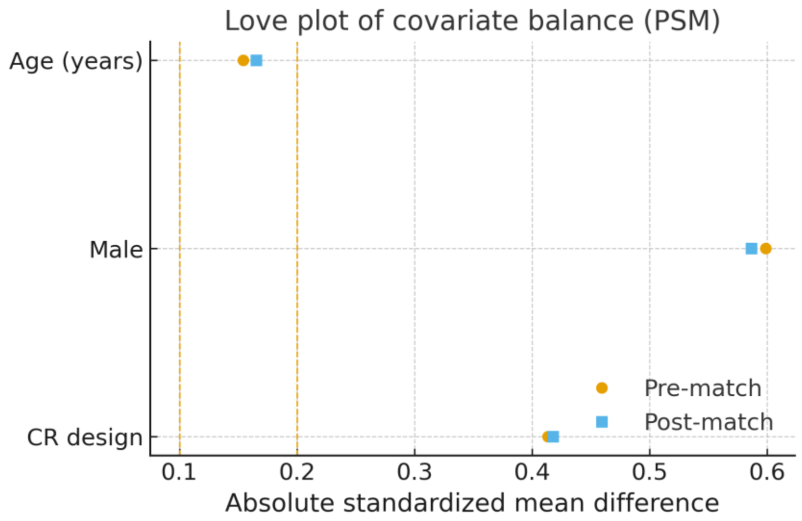

Figure S1. Love plot of covariate balance before and after 1:1 propensity score matching (age, sex, implant design [CR]). Dashed vertical lines at 0.10 and 0.20 denote common balance thresholds.

Supplementary Table S1. Standardized peri-operative protocol common to cemented and cementless TKA.

Domain	Protocol step	Cemented	Cementless	Notes
Pre-operative pathway	Indication and optimization	✓	✓	
Anesthesia	Regional anesthesia per institutional pathway	✓	✓	
Antibiotic prophylaxis	Administered per institutional protocol	✓	✓	
Tourniquet – application	Applied prior to incision	✓	✓	
Tourniquet – during fixation	Re-inflation during cement pressurization	As per surgeon preference	Not applicable	Only cemented cases involved re-inflation for cement pressurization.
Hemostasis adjuncts	Standard intra-operative hemostasis per pathway	✓	✓	No patient-level suction data available.
Tranexamic acid (TXA)	Administered per institutional PBM protocol	✓	✓	
Analgesia	Multimodal analgesia per pathway	✓	✓	
Thromboprophylaxis	Pharmacologic and mechanical	✓	✓	

	prophylaxis per institutional protocol			
Wound management	Standard closure and dressing	✓	✓	
Post-operative Hb monitoring	Hb measured pre-op, POD1, POD3, POD5	✓	✓	Timepoints correspond to primary analysis.
Transfusion policy	Per institutional PBM framework	✓	✓	
Discharge pathway	Standard mobilization and discharge criteria	✓	✓	

*PBM, patient blood management; POD, postoperative day; TKA, total knee arthroplasty; TXA, tranexamic acid.
